# A needs-assessment survey of the high school LGBTQ+ environment by a health science center interprofessional team

**DOI:** 10.3389/fsoc.2024.1356007

**Published:** 2024-08-22

**Authors:** Rafael Velasquez, Mary E. Moore, Gabrielle Sheets, Christian Nieves-Rivera, Sonya Van Nuland, Martha Cuccia, Fern Tsien, Andrew D. Hollenbach

**Affiliations:** ^1^Ochsner Medical Center, New Orleans, LA, United States; ^2^Stritch School of Medicine, Loyola University Chicago, Chicago, IL, United States; ^3^School of Graduate Studies, Louisiana State University Health Sciences Center, New Orleans, LA, United States; ^4^Children’s Hospital of New Orleans, New Orleans, LA, United States; ^5^School of Medicine, Louisiana State University Health Sciences Center, New Orleans, LA, United States; ^6^School of Public Health, LSU Health Sciences Center, New Orleans, LA, United States

**Keywords:** LGBTQ+, LGBTQ+ advocacy, LGBTQ+ school environment, high school, outreach, LGBTQ+ mental health, school health, adolescent health

## Abstract

Despite improvements in the awareness and acceptance of lesbian, gay, bisexual, transgender, queer, and other sexual and gender diverse (LGBTQ+) individuals, the LGBTQ+ community continues to experience discrimination, which can result in adverse health outcomes. In particular, LGBTQ+ youth have an increased risk of experiencing depression, substance abuse, and suicide. Societal stigma and rejection, bullying, and familial disapproval all contribute to these health disparities. In recognition of these inequities, an interprofessional team of biomedical faculty members, staff, and trainees from the Louisiana State University Health Science Center (LSUHSC) in New Orleans developed the needs-assessment evaluation, the Gender and Sexual Minority Youth Outreach Survey (GSMYO) for high school students. Health science centers have access to resources and experienced personnel who can provide support and education to high school students, teachers, and administrative staff. However, it is important to first determine the high schools’ specific needs, attitudes towards LGBTQ+ acceptance, and their current resources. Faculty, staff, and trainees from the LSUHSC Science Youth Initiative (SYI) and the LSUHSC LGBTQ+ Organization, Tiger Pride, administered the short, anonymous survey to adolescents attending Southeast Louisiana high schools. English Language Learner (ELL) students received the survey in Spanish. Results from the GSMYO needs-assessment survey are presented. Other health science centers may adapt the presented survey to develop needs-based LGBTQ+ high school programs to address the educational and health inequities in their own communities, regardless of location or demographic region.

## 1 Introduction

The National Institutes of Health officially recently designated lesbian, gay, bisexual, transgender, queer and other sexual and gender diverse (LGBTQ+) individuals as a health disparity population (National Heart, Lung, and Blood Institute & National Institutes of Health, 2014; Dankwa-Mullan and Ṕerez-Stable, 2016; Eliscu et al., 2023). Compared to heterosexual and gender binary peers, LGBTQ+ individuals are more than 2.5 times more likely to experience anxiety, depression, higher rates of suicidal thoughts, negative health behaviors (e.g., alcohol and tobacco use, physical inactivity, obesity), and sexually transmitted infections (Cochran et al., 2003; Committee on Lesbian, Gay, Bisexual, and Transgender Health Issues and Research Gaps and Opportunities, and Board of the Health of Select Populations, 2011; Buchting et al., 2018; Shover et al., 2018; Herman et al., 2019; Eliscu et al., 2023). Recent reports state that 81% of transgender adults in the U.S. have thought about suicide, 42% of transgender adults have attempted it, and 56% have engaged in non-suicidal self-injury over their lifetimes (Kidd et al., 2023).

LGBTQ+ youth, in particular, face discrimination, harassment, family & social rejection, or violence, which lead to an increased risk of depression, substance abuse, and suicide (McConnell et al., 2015; Mustanski et al., 2015; Gonzales and Rendon-Garcia, 2016; Hatzenbuehler and Pachankis, 2016; Gonzales and Henning-Smith, 2017; Centers for Disease Control and Prevention National Center, 2021). The Centers for Disease Control and Prevention (CDC)’s Youth Risk Behavior Surveillance System (YRBSS) monitors health among 9th through 12th grade students in the U.S. (Kann et al., 2018; Center for Disease Control and Prevention, 2019). The YRBSS reports that 69% of LGBQ+ youth feel persistently depressed and hopeless, compared to 35% of heterosexual youth (Center for Disease Control and Prevention, 2019; Szucs et al., 2022). LGBQ+ youth consistently report higher rates of bullying, being threatened or injured with a weapon, and dating violence (Center for Disease Control and Prevention, 2019; Szucs et al., 2022). During the COVID-19 pandemic, LGBQ+ students were four times more likely to attempt suicide, and 20% of LGBQ+ students said they had been physically abused by a parent or other adult at home compared to 10% of heterosexual students (Szucs et al., 2022). Data indicate that 82% of transgender and gender nonconforming (TGNC) individuals have considered killing themselves and 40% have attempted suicide, compared to the U.S. frequencies of 12.1 and 4.1%, respectively (Grossman et al., 2016; Austin et al., 2022).

Research shows that students who feel supported by their school community do better socially, emotionally, and academically (Ybarra et al., 2015; Gegenfurtner and Gebhardt, 2017; Center for Disease Control and Prevention, 2019; Wilkins et al., 2019). When schools implement LGBTQ+-supportive policies and practices, all students experience less emotional distress, violence, harassment, and suicidal behaviors (Gegenfurtner and Gebhardt, 2017; Center for Disease Control and Prevention, 2019; Lessard et al., 2020; Kaczkowski et al., 2022). The CDC’s “What Works in Schools” program has identified inclusive practices that benefit both LGBTQ+ students and their heterosexual peers (Centers for Disease Control and Prevention, 2023). However, some schools are resistant to these implementations, so students may not have allies or adults they can trust (Kaczkowski et al., 2022; Shattuck et al., 2022). In fact, LGBTQ+ youth are more likely to miss school because of safety concerns (Gegenfurtner and Gebhardt, 2017; Center for Disease Control and Prevention, 2019; Szucs et al., 2022). Studies recommend training of teachers, counselors, social workers, and mental health practitioners to systematically incorporate knowledge and evidence-based practices to enable them to become culturally and clinically skilled in working with LGBTQ+ and TGNC youth and their families (Grossman et al., 2016; Gegenfurtner and Gebhardt, 2017; Phelan et al., 2017).

Since the NIH designated LGBTQ+ as a health disparity population, health care organizations and academic institutions have created guidelines to reduce these inequalities (Dankwa-Mullan and Ṕerez-Stable, 2016; Phelan et al., 2017; Rafferty and AAP Committee on Psychosocial Aspects of Child and Family Health, AAP Committee on Adolescence, AAP Section on Lesbian, Gay Bisexual, and Transgender Health and Wellness, 2018; Cronin and Stockdale, 2021; American Academy of Family Physicians, 2023; Eliscu et al., 2023). The Liaison Committee on Medical Education now includes community service opportunities in their accreditation guidelines, and many graduate programs in the biomedical sciences and public health incorporate service learning as part of their curriculum (Woodhouse et al., 2006; Sabo et al., 2015; Loh et al., 2016; Phelan et al., 2017; Abraham and Torner, 2021). Studies describe a dramatic shift in the medical school curriculum to reduce medical student biases toward LGBTQ+ individuals by increasing opportunities to provide care to sexual minorities, an improved diversity climate, and more favorable interaction with sexual minorities (Phelan et al., 2017). These programs provide valuable opportunities for trainees in health care professions, biomedical research, and public health to strengthen community-campus partnerships and address health disparities in vulnerable populations (Seifer, 1998; Blue et al., 2006; Woodhouse et al., 2006; Sheridan et al., 2010; Brazeau et al., 2011; Sabo et al., 2015; Loh et al., 2016; Phelan et al., 2017; Abraham and Torner, 2021).

The goal of the present study is to conduct a needs-assessment among high school students residing in Southeastern Louisiana. Results of the survey will be used to develop needs-based LGBTQ+ health equity initiatives that improve LGBTQ+ education, improve training of emerging health care and public health professionals, increase accessibility to preventative health services, and reduce LGBTQ+ health disparities.

## 2 The interprofessional team

The Louisiana State University Health Sciences Center (LSUHSC) in New Orleans, LA is comprised of six schools (Medicine, Graduate Studies, Public Health, Nursing, Allied Health Professions, and Dentistry). In keeping with national guidelines, LSUHSC provides health education service learning to high schools while allowing future health care professionals and academic trainees to develop a sense of commitment to their communities (Gunaldo et al., 2018, 2020). For more than 20 years, LSUHSC in New Orleans has received federal, local, and foundational funding to support educational pipeline/pathway programs including the Science Youth Initiative (SYI) (Erickson et al., 2022; Sims et al., 2022; Hess et al., 2023; Moore et al., 2024). During the academic year (August–May), an interprofessional team of LSUHSC faculty, staff, and trainees teach high school students via practical experiments to enhance their science curriculum, improve classroom/standardized test scores, and provide career guidance. The curricula were developed in partnership with teachers from more than 50 schools throughout Southeastern Louisiana. Anonymous formative and summative evaluations by participants have measured satisfaction of the program, demographics, learning gains, topic retention, and long-term tracking of career plans (Erickson et al., 2022; Sims et al., 2022; Hess et al., 2023; Moore et al., 2024).

All LSUHSC faculty, trainees, and staff are required to complete yearly online training covering how to prevent harassment and discrimination. These topics are reinforced during in-person training which includes workshops on microaggressions, implicit bias, and SafeZone Training (Bolger and Killerman, 2018; Harrison-Bernard et al., 2020; Shattuck et al., 2022). SafeZone Training develops, enhances and maintains safe environments in workplaces, schools and other social settings that support LGBTQ+ individuals, as well as straight, cisgender people (Bolger and Killerman, 2018; Harrison-Bernard et al., 2020; Shattuck et al., 2022). Workshops include videos, seminars, interactive activities, and panel discussions, and are facilitated by diversity education experts at the institutional, state, and national level. Additionally, all LSUHSC adults teaching students under the age of 18 require a criminal background check, fingerprinting, and the completion of an online training module by the Louisiana Department of Children and Family Services.

## 3 The gender and sexual minority youth outreach survey

To find out how to help our communities, it was first necessary to evaluate the needs of individual high schools and to determine whether these needs varied depending on the location, nature, and composition of the student population. Therefore, faculty directors of the SYI created the Gender and Sexual Minority Youth Outreach Survey (GSMYOS), modeled after the CDC’s Youth Risk Behavior Surveillance System (YRBSS) high school questionnaire, (Centers for Disease Control and Prevention National Center, 2021). The GSMYOS is a short, simple, anonymous survey asking adolescents to rate their high school’s attitudes towards LGBTQ+ acceptance and available resources with the overall goal of developing needs-based educational and support programs led by health care professional trainees and faculty for LGBTQ+ youth. The survey was approved by the Institutional Review Board and all participants had parent/guardian consent.

### 3.1 Survey administration

Data were collected from SYI workshop participants enrolled in high schools from Southeastern Louisiana including public (ELL and non-ELL) and private (Catholic and non-Catholic) schools in urban and suburban regions of a wide range of socioeconomic backgrounds and racial and ethnic groups. LSUHSC facilitators supervised the in-person survey, in which students seated themselves separately from their peers to ensure privacy. Upon completion, surveys were collected in an envelope to maintain anonymity. Students were informed that the survey was completely anonymous and voluntary and that a blank (incomplete) survey form would be accepted. Adolescents enrolled in English Language Learner (ELL) programs whose first language was Spanish received the survey translated to their native language.

### 3.2 Analysis

The data were analyzed using the following methods: For questions using a 5-point Likert scale (i.e., strongly disagree = 1 to strongly agree = 5), the data were grouped according to school type (private Catholic, private non-Catholic, public), if students were enrolled in an ELL program, and by school location (suburban, urban). Mean and standard error were computed for each category, and a two-sample *t*-test was used to determine significance in answers based on school type and school location. A significance threshold was set at *α* = 0.05. Since the responses for Statement 2 were not on a scale; responses were analyzed using a Chi-Square test. Significance threshold was set at *α* = 0.05.

### 3.3 Demographics

**Figure 1:**
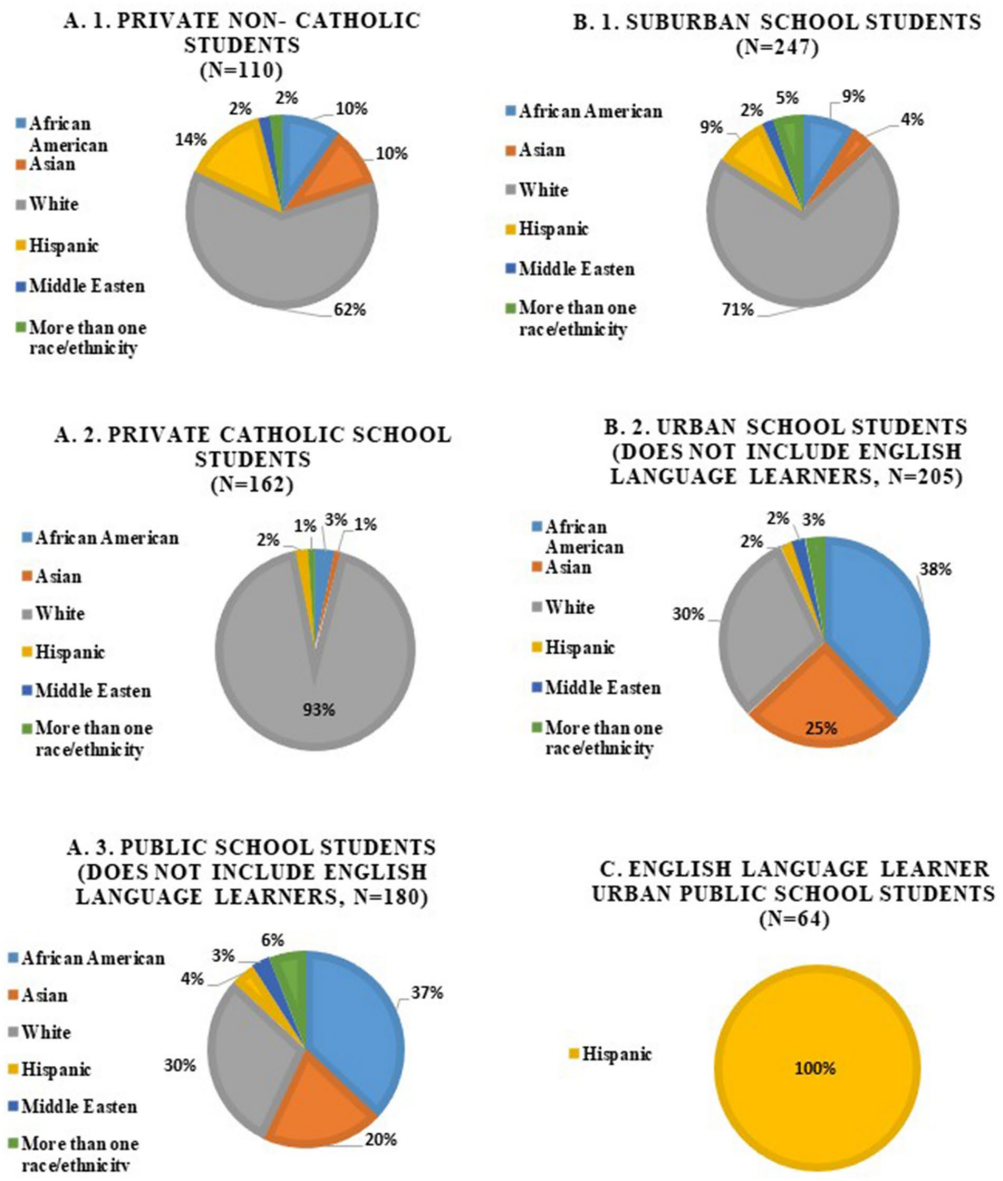
Demographics of high school students who completed the Gender and Sexual Minority Youth Outreach Survey needs-assessment (*n* = 516).

A total of 516 high school students were surveyed from seven different schools; one urban school had both ELL and non-ELL students, but their results were analyzed separately. The demographics regarding race varied, ranging from private Catholic schools with a predominantly White student body to public schools with a more racially diverse population (Figure 1). Furthermore, higher levels of diversity were observed in urban schools relative to suburban schools.

### 3.4 Survey question results

**Table 1:** Summary of high school student responses to the Gender and Sexual Minority Youth Outreach needs-assessment survey.

Statement 1: I feel that my school is an LGBTQ+ friendly environment.								
Percentages by school type					Percentages by school location			
Likert scale	Private Non-Catholic (*n* = 110)	Private Catholic (*n* = 162)	Public Non-ELL (*n* = 180)	Public ELL (*n* = 64)	Likert scale	Suburban (*n* = 247)	Urban Non-ELL (*n* = 205)	Urban ELL (*n* = 64)
Strongly disagree (1)	2.8%	9.3%	5.0%	6.3%	Strongly disagree (1)	6.8%	4.3%	6.3%
Disagree (2)	2.8%	36.6%	6.1%	12.5%	Disagree (2)	21.6%	4.3%	12.5%
Neutral (3)	22.9%	40.4%	18.9%	35.9%	Neutral (3)	33.9%	13.6%	**35.9%**
Agree (4)	53.2%	10.6%	35.0%	26.6%	Agree (4)	29.0%	34.3%	26.6%
Strongly agree (5)	18.3%	3.1%	35.0%	18.8%	Strongly agree (5)	8.7%	43.6%	18.8%

Statement 2: I personally know someone who identifies as LGBTQ+.								
Percentages by school type					Percentages by school location			
Likert scale	Private Non-Catholic	Private Catholic	Public Non-ELL	Public ELL	Likert scale	Suburban	Urban Non-ELL	Urban ELL
Yes (1)	90.8%	91.4%	92.8%	34.4%	Yes (1)	90.4%	95.0%	34.4%
No (2)	1.8%	6.8%	2.8%	42.2%	No (2)	4.8%	2.1%	42.2%
I do not know (3)	7.3%	1.9%	3.9%	18.8%	I do not know (3)	4.8%	2.1%	18.8%
Do not care to respond (4)	0.0%	0.0%	0.6%	4.7%	Do not care to respond (4)	0.0%	0.7%	4.7%

Statement 3: I have witnessed bullying (verbal, physical, online, etc.) of classmates openly identified or perceived to be LGBTQ+.								
Percentages by school type					Percentages by school location			
Likert scale	Private Non-Catholic	Private Catholic	Public Non-ELL	Public ELL	Likert scale	Suburban	Urban Non-ELL	Urban ELL
Strongly disagree (1)	14.5%	11.1%	15.0%	15.9%	Strongly disagree (1)	11.9%	17.1%	15.9%
Disagree (2)	29.1%	30.2%	34.4%	28.6%	Disagree (2)	29.2%	37.1%	28.6%
Neutral (3)	20.9%	21.0%	20.0%	34.9%	Neutral (3)	21.5%	18.6%	34.9%
Agree (4)	34.5%	35.2%	26.7%	15.9%	Agree (4)	35.3%	23.6%	15.9%
Strongly agree (5)	0.9%	2.5%	3.9%	4.8%	Strongly agree (5)	2.2%	3.6%	4.8%

Statement 4: I have heard others in school speak about LGBTQ+ matters in a negative context asked students about bullying on their campuses.								
Percentages by school type					Percentages by school location			
Likert scale	Private Non-Catholic	Private, Catholic	Public Non-ELL	Public ELL	Likert scale	Suburban	Urban Non-ELL	Urban ELL
Strongly disagree (1)	5.5%	1.9%	10.6%	15.9%	Strongly disagree (1)	3.9%	11.4%	15.9%
Disagree (2)	3.7%	11.7%	15.0%	28.6%	Disagree (2)	8.4%	17.1%	28.6%
Neutral (3)	15.6%	18.5%	19.4%	34.9%	Neutral (3)	17.7%	19.3%	34.9%
Agree (4)	49.5%	52.5%	45.0%	15.9%	Agree (4)	50.2%	45.7%	15.9%
Strongly agree (5)	25.7%	15.4%	10.0%	4.8%	Strongly agree (5)	19.9%	6.4%	4.8%

ELL, English Language Learner; Total number of student responses: *n* = 516.

#### 3.4.1 Question 1: I feel that my campus is an LGBTQ+-friendly environment

Survey question results are summarized in Table 1. Responses to this statement varied significantly by school type:

- Private non-Catholic schools (*n* = 110) and non-ELL public schools (*n* = 180) agreed (53.2, 35% respectively). Using the Likert scale (strongly disagree = 1, strongly agree = 5) their means were similar, 3.78 and 3.88, respectively (*p* = 1.97).
- Students from private Catholic schools (*n* = 162) were the most likely to disagree (36.6%) with this statement. The average response from students in this category (2.6) is significantly lowest, indicating that students in private Catholic schools do not consider their school as LGBTQ+-friendly.
- The majority of ELL students attending public Schools (public ELL) chose neutral (35.9%), with a mean of 3.39.
- All schools’ means were significantly higher than private Catholic schools.

Significant differences were seen based on location:

- Although the majority of students from all tested locations agreed that their school was LGBTQ+ friendly (suburban, urban non-ELL, and urban ELL student average scores were 3.09, 4.08, and 3.39, respectively).
- Urban non-ELL students (*n* = 205) were most likely to strongly agree (43.6%).
- The majority of the students in suburban and urban ELL remained neutral.

#### 3.4.2 Question 2: Do you personally know someone who openly identifies as LGBTQ+?

- The vast majority of non-ELL public school, private Catholic school, and private non-Catholic school students (92.8, 91.3, and 90.8%) or suburban and non-ELL urban (95.0 and 90.4%) answered that they knew someone who openly identified as LGBTQ+.
- However, the answers of public ELL students were significantly different from other student types. The majority of ELL students reported that they did not know someone who identified as LGBTQ+ (42.2%).

#### 3.4.3 Statement 3: I have witnessed bullying (verbal, physical, online, etc.) of classmates openly identified or perceived to be LGBTQ+

We observed no statistical difference in the response of students by school type:

- Average responses ranged from 2.6 to 2.8.
- Public ELL students had a higher percentage of “neutral” responses (34.9%).

In contrast, statistically significant differences were found when the data were evaluated by location:

- A larger percentage of students from Suburban schools “agreed” or “strongly agreed” to have witnessed bullying (35.3%) than other locations.
- Non-ELL urban students reported the least amount of bullying relative to suburban students (non-ELL urban 17.1% strongly disagree, 37.1% disagree; suburban 11.9% strongly disagree, 29.9% disagree).
- Responses for urban ELL students also differed from suburban schools with the differences approaching statistical significance (*p* = 0.06).

#### 3.4.4 Question 4: I have heard others in school speak about LGBTQ+ matters in a negative context

- Students from private non-Catholic schools and private Catholic schools had heard about LGBTQ+ matters in a negative context (with means of 3.8 and 3.7, respectively; and “agreed” or “strongly agreed” 75 and 67.9% respectively).
- In contrast, ELL students from public schools had the lowest mean out of all school types (2.7) and the highest percentage (44.5%) of students saying that they had not heard about LGBTQ+ matters being discussed in a negative context. The majority of ELL students stayed neutral (34.9%).
- These statistics are significantly different when compared to other school categories, and even differs from Non-ELL public school students, whose mean was 3.3.

When the responses for this statement were analyzed by location:

- Students from suburban schools had the highest average (3.7), followed by non-ELL urban (3.2) and then urban ELL students (2.6).
- Of note, the responses of suburban students were clustered at the “agree” (50.2%) and “strongly agree” (19.9%), with the majority of ELL students as staying neutral (34.9%).

## 4 Discussion

Our results provide insights into the environments that LGBTQ+ students encounter at their respective schools in Southeastern Louisiana. We found that schools in suburban areas, although equally as likely as their non-ELL urban counterparts to know an LGBTQ+ person, reported lower levels of LGBTQ+ friendliness in their school and higher levels of negative comments and bullying. This result indicates that although many suburban students are aware of LGBTQ+ individuals, there also exists higher levels of intolerance and hostility towards this population.

The responses of ELL students in public schools differed from non-ELL students. ELL students were unique in that they were less likely than their non-ELL counterparts to report that their school was LGBTQ+ friendly and less likely to know someone who identified as LGBTQ+. They reported, however, fewer incidents of negative remarks and witnessing bullying than non-ELL students. One possible explanation is that many ELL students are raised in cultures and environments in which discussion of LGBTQ+ issues are considered a taboo topic (Dumas, 2008; Witcher, 2014; Gray et al., 2015; Quidley-Rodriguez and Gattamorta, 2019). Furthermore, they may wish to assimilate, in which case their comments may reflect an attempt to fit into their new environment (Gray et al., 2015; Quidley-Rodriguez and Gattamorta, 2019). ELL educators often find themselves not only teaching language, but also American culture, so guidelines have been proposed to embed LGBTQ+ cultural lessons in their classroom environment (Dumas, 2008; Witcher, 2014). ELL teachers are recommended to reframe classroom discussions to include LGBTQ+ content, and in particular acceptance of transgender individuals (Dumas, 2008; Witcher, 2014; Grossman et al., 2016; Shariff-Marco et al., 2017).

Private non-Catholic and non-ELL public school students were the most likely to report that their school is LGBTQ+ friendly, while private Catholic school students were the least likely. Furthermore, while students from all school types were equally likely to have witnessed bullying, students from private Catholic schools and private non-Catholic schools were the most likely to have heard negative remarks about the LGBTQ+ community. According to scholars, the opportunities to include the LGBTQ+ community in Catholic education aligns with tenets of Catholic Social Teaching (the life and dignity of the person, the preference for the vulnerable, and the common good) (United States Conference of Catholic Bishops, 2005; Huchting and Fisher, 2019). However, a hostile climate exists for LGBTQ+ students, and the message of inclusion is contradicted when LGBTQ+ students experience microaggressions (Huchting and Fisher, 2019; Janoski, 2023). Extensive research by Maher et al. demonstrates the connections between religiosity, masculinity, and intolerance in Catholic schools (Maher, 2007; Maher and Sever, 2007; Maher et al., 2008; Maher, 2013). Catholic secondary school staff and administrators’ recommendations for promoting a more inclusive LGBTQ+ environment include (1) exposure to diverse perspectives and world views, (2) a “more personal, socially conscious, socially compassionate” learning environment, (3) professional development, (4) and the creation of ally and affinity groups (Maher, 2007; Maher and Sever, 2007; Maher et al., 2008; Maher, 2013; Callaghan, 2016; Huchting and Fisher, 2019; Parodi-Brown, 2019). Studies report that Catholic students are more accepting towards LGBTQ+ individuals when they had more personal experiences with them (Maher, 2007; Maher and Sever, 2007; Maher et al., 2008; Callegher, 2010; Callaghan, 2016; Huchting and Fisher, 2019). The Catholic Pastoral Committee on Sexual Minorities spearheaded the Safe Schools Initiative, based on a “training the trainer” model implemented in some U.S. Catholic schools (Bayly, 2013; Ratts et al., 2013).

## 5 Future directions

Our needs-assessment results provide valuable information for the development of health science center-led outreach programs to provide (1) education about LGBTQ+ issues to all high school students, (2) support for students who identify as LGBTQ+, and (3) service learning opportunities for health science center trainees. While all high schools would benefit from a centralized Core Curriculum, each school has its own unique environment that would require specific modules to address their individual needs. Therefore, based on our results, we propose supplementary modules targeting specific school environments. All of these would be led by health science center trainees, faculty, and staff. In addition, there are a variety of online resources that can aid in the development of the overarching program and modules including the Gay, Lesbian & Straight Education Network (GLSEN) Safe Space Kit (GLSEN, 2016), and the U. S. Department of Health and Human Services (Stopbullying.gov, 2021). One limitation of this study includes the lack of sex or gender identity in our participants but will be included in the future.

## Data Availability

The original contributions presented in the study are included in the article; further inquiries can be directed to the corresponding author.
